# Progress and Prospects of Gene Editing in Pluripotent Stem Cells

**DOI:** 10.3390/biomedicines11082168

**Published:** 2023-08-01

**Authors:** Zhenwu Zhang, Xinyu Bao, Chao-Po Lin

**Affiliations:** School of Life Science and Technology, ShanghaiTech University, Shanghai 201210, China; zhangzhw2@shanghaitech.edu.cn (Z.Z.); baoxy2022@shanghaitech.edu.cn (X.B.)

**Keywords:** pluripotent stem cell, induced pluripotent stem cell, CRISPR-Cas9, base editor, prime editor, gene editing

## Abstract

Applying programmable nucleases in gene editing has greatly shaped current research in basic biology and clinical translation. Gene editing in human pluripotent stem cells (PSCs), including embryonic stem cells (ESCs) and induced pluripotent stem cells (iPSCs), is highly relevant to clinical cell therapy and thus should be examined with particular caution. First, since all mutations in PSCs will be carried to all their progenies, off-target edits of editors will be amplified. Second, due to the hypersensitivity of PSCs to DNA damage, double-strand breaks (DSBs) made by gene editing could lead to low editing efficiency and the enrichment of cell populations with defective genomic safeguards. In this regard, DSB-independent gene editing tools, such as base editors and prime editors, are favored due to their nature to avoid these consequences. With more understanding of the microbial world, new systems, such as Cas-related nucleases, transposons, and recombinases, are also expanding the toolbox for gene editing. In this review, we discuss current applications of programmable nucleases in PSCs for gene editing, the efforts researchers have made to optimize these systems, as well as new tools that can be potentially employed for differentiation modeling and therapeutic applications.

## 1. Introduction

Pluripotent stem cells (PSCs) possess two unique characteristics, indefinite self-renewal and the potential to differentiate into nearly all cell types of three germ layers, therefore holding great promise for regenerative medicine. The two major types of PSCs are embryonic stem cells (ESCs) derived from the inner cell mass of the preimplantation embryo [1] and induced pluripotent stem cells (iPSCs) generated by reprogramming of somatic cells [2,3]. With technical advances in the past decade, human iPSCs can now be generated by RNA viruses, episomal vectors, or chemical cocktails, avoiding genomic integrations [4,5,6,7,8,9]. Compared with human ESCs, iPSCs are generated from autologous cells and are easier to obtain, enabling iPSCs to be applied in cell-based therapies [10,11,12]. For example, mutations of patient-derived iPSCs can be corrected and differentiated towards specific cell types for therapeutic purposes [13,14]. For cancer immunotherapy, autologous or immune-compatible iPSCs can be modified (e.g., introducing chimeric antigen receptors) and serve as unlimited, “off-the-shelf” sources of engineered immune cells [15,16]. 

**Figure 1:**
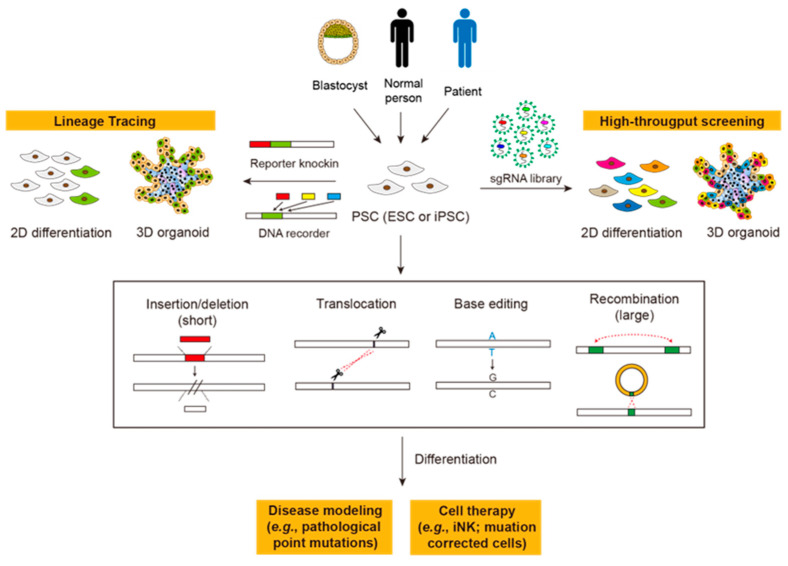
Gene editing in PSC-based basic research and clinical applications. ESC, embryonic stem cell; iPSC, induced pluripotent stem cell; iNK, iPSC-derived NK cells. See the text for details.

In addition to clinical purposes, PSCs also possess great value in basic research [17]. PSCs are long-standing models for investigating determinants or modulators of lineage specification or terminal differentiation. The advent of organoid cultures further expanded PSC applications [18,19]. Organoids are self-organized cell aggregates that recapitulate cellular compositions and organizations of corresponding tissues, and have been widely employed as in vitro models to study tissue development or diseases [20,21,22,23,24]. PSC-based in vitro differentiation models in 2D and 3D conditions are also platforms for high-throughput screening [25,26] or lineage tracing [27,28], serving as precious tools for studying human-specific development or diseases lacking appropriate mouse models (Figure 1).

Many of those aforementioned applications require gene editing in PSCs. A major merit of performing gene editing in PSCs is the high stability in both the genome and the cell fate potential. Thus, the integrity of the genome of engineered PSCs can be thoroughly examined before any application. Recently, the ability to edit the genome of PSCs has been greatly elevated with the development of gene editing tools, especially programmable nucleases. The ease of use and the high editing efficiency of programmable nucleases greatly facilitate the applications of PSCs in basic research, such as knock-out/knock-in, disease modeling, and correction of genetic mutations [29,30,31]. Nonetheless, gene editing in PSCs also requires careful evaluation due to their spectacular properties: comparing with adult (stem) cells which have limited longevity, PSCs can propagate almost indefinitely and pass all mutations to their progenies, making the preciseness of gene editing in PSCs of great concern. This concern is further exacerbated with recent studies demonstrating that gene editing mediated by CRISPR associated protein 9 (Cas9)-induced DNA cleavage in human PSCs produces genome-wide mutations and rearrangements [32,33]. Moreover, the selection of successfully edited PSC clones also favors the accumulation of p53 mutations, hampering further utilization of this powerful technology in human PSCs [33,34]. Here, we review the current progress and prospects of gene editing, which we define as inducing permanent changes on DNA sequences, in PSCs and the efforts researchers have made to optimize those tools (Table 1).

## 2. DSB-Mediated Gene Editing by Programmable Nucleases

Since late 1980, homologous recombination (HR) has been widely employed for genome editing in mouse ESCs to create genetically modified mice, establishing a paradigm for studying gene functions, disease mechanisms, and lineage specifications [35]. In a general protocol, donor DNAs with homologous arms are electroporated to mouse ESCs, and the HR-mediated editing (knock-out or knock-in) occurs spontaneously at very low frequencies [36]. This laborious and time-consuming procedure was changed by groundbreaking works of the Haber and Jasin groups, demonstrating that the induction of double-strand breaks (DSBs) by endonucleases at sites aimed to be edited could trigger DNA repair pathways and dramatically increase the efficiencies of HR in yeasts and mammalian cells [37,38]. On the other hand, repair of DSBs by non-homologues end joining (NHEJ) can generate insertions/deletions (indels), ablating the protein expression or function. These findings inspired researchers to look for programmable nucleases that can induce DSBs at desired sites. To date, zinc finger nucleases (ZFNs), transcription activator-like effector nucleases (TALENs), and CRISPR-Cas systems are the most frequently employed methods. 

Different cells exhibit distinct sensitivities to DSBs. PSCs, as derived from early embryos, have evolved at least two mechanisms to keep low DNA mutation rates compared to somatic cells, thus protecting the genome integrity from the accumulation of genetic mutations. First, PSCs possess a superior ability to repair DNA damages by expressing abundant mismatch repair proteins or avoiding error-prone repair pathways [39,40,41,42]. The high DNA repair capability also suggests the fast removal of the DSB marker γ-H2AX, thus antagonizing its association with apoptosis-inducing factor (AIF) for the formation of the “degradosome” that leads to chromatin remodeling and large-scale DNA fragmentation [43,44]. Second, once the level of DNA damage accumulates over a threshold, PSCs are highly prone to apoptosis, eliminating themselves from the whole population. Those self-protection mechanisms of PSCs are double-edged swords for applying those powerful DSB-dependent gene editing tools in PSCs, since the sensitivity of PSCs toward DNA damages could lead to the low editing efficiency or undesired loss/mutation of genomic safeguards [32,33]. Here, we briefly review those three DSB-dependent programmable nucleases and their off-target effects in the context of PSCs, as their development and mechanisms have been extensively reviewed [30,45,46,47,48,49].

### 2.1. Zinc-Finger Nuclease (ZFN)

Zinc-finger nucleases (ZFNs) are artificially engineered endonucleases that recognize specific DNA sequences by customized zinc-finger protein arrays [50,51]. The zinc finger domain of a ZFN, which binds to a specific DNA sequence, is fused with the FokI nuclease. With a pair of ZFNs binding to target sites of genome DNA in the opposite orientation, FokI will be dimerized and produce DNA DSBs that strongly activate the DNA repair pathway and greatly increase gene editing efficiencies compared with the natural recombination rate in the absence of DNA breaks. NHEJ or homology-directed repair (HDR) of these DSBs will lead to random indels or sequence replacement, which can be utilized for gene knock-out or knock-in, respectively [52]. Recently, new dimer architectures, made possible by different linkers between zinc finger proteins and FokI, were successfully developed and greatly increased the design flexibility [53]. Currently, several ZFN-based gene- and cell-therapies developed by Sangamo Therapeutics are under clinical trials.

ZFNs-mediated genetic manipulation in human PSCs was first reported in 2007. The ZFN and the donor DNA were delivered to human ESCs by lentiviruses to knock-in a green fluorescent protein (GFP)-expressing cassette to the end of the *CCR5* gene through HDR with a 5.3% efficiency [54]. ZFN was also used to disrupt the *PIG-A* gene in human ESCs and iPSCs [55]. Hockemeyer et al. used ZFNs to tag *EGFP* to *PITX3* or insert *EGFP* to the safe harbor locus, *AAVS1*, to generate reporter or drug-inducible cell lines in human ESCs [56]. These pioneer studies demonstrated the superior efficiencies of DSB-activated recombination in PSCs.

#### On- and Off-Targeting of ZFNs

With the high genome editing efficiency, the specificity of the programmable nucleases also came into view. The on-target efficiency of nucleases can be examined by Sanger/high-throughput sequencing or mismatch-sensitive enzymes such as T7 endonuclease I (T7E1) [57,58]. Nonetheless, the evaluation of off-target effects is not as trivial as it appears to be: high-throughput transcriptomic sequencing (RNA-seq) is able to detect mutations on coding and non-coding genes with good coverage, yet can neither determine whether mutations happen on DNA or RNA, nor reveal gene duplications, chromosome translocations, and mutations in regulatory regions [59]. The whole-genome/exome sequencing (WGS/WES) has been applied to examine the ZFN-mediated correction of a point mutation on the *A1AT* site in human iPSCs [60]. Nonetheless, although the WGS is a reliable way to measure off-target edits in single cell-derived colonies, it is not suitable for measuring the rate of off-target editing at the population level, as the mutation frequencies at off-target sites are too low to be detected by typical sequencing depth.

To resolve these issues, other methods have been developed to enhance the sensitivity of off-target measurement in an unbiased, genome-wide manner. For example, the in vitro selection and the integrase-deficient lentivirus (IDLV) capture were developed to examine the ZFN cleavage sites [61]. The former uses DNA substrate libraries to determine the specificity of nucleases in vitro [61], while the latter uses IDLV to integrate at cleavage sites and detect off-target edits in cells [62]. Both methods were employed to examine the off-target effects of a *CCR5*-targeting ZFN and uncovered a previously known off-target site, *CCR2* [61,62,63,64]. In addition to the *CCR2* site, in vitro selection also identified large numbers of off-targets from the substrate library, some of which can be identified in cells [61]. Together, these studies suggest that the off-target effects of ZFNs should be meticulously evaluated. As various methods have been developed for measuring CRISPR-Cas9 off-targets (see later), the specificity of ZFNs can be systematically addressed.

### 2.2. Transcription Activator-like Effector Nucleases (TALENs)

The transcription activator-like effector nuclease (TALEN) is composed of the DNA binding domain and the DNA cutting domain [65,66]. The DNA binding domain, derived from the transcription activator-like effector (TALE) protein in the *Flavobacterium*, contains multiple tandem repeats of 33–34 amino acids with divergent dual residues at positions 12 and 13 (repeat variable di-residue, or RVD), which determine TALE’s binding specificity. The DNA cutting domain is the cleavage domain of FokI endonuclease. Thus, both ZFNs and TALENs can be viewed as FokI endonucleases targeted by engineered proteins that recognize specific DNA sequences. Similar to ZFNs, TALENs were also introduced into cells for genome editing, making knock-out, knock-in, and site-specific mutations [66,67]. One advantage of TALENs for researchers is that all the RVDs and their recognized sequences are open resources, while ZFNs are only available from Sangamo Therapeutics, Richmond, CA, USA. So far, there have been several TALEN-based gene- and cell-therapies under clinical trials [68,69].

In PSCs, TALENs have been used for genome editing in iPSCs generated from dermal fibroblasts in MELAS (myopathy, encephalopathy, lactic acidosis, and stroke-like episodes) patients with mitochondrial G13513A mutation [70]. Moreover, genes associated with human cardiovascular diseases, such as *TNNT2*, *LMNA/C*, *TBX5*, *MYH7*, *ANKRD1*, and *NKX2.5*, were also knocked-out by TALENs in human iPSCs to build cardiovascular disease models [71]. X-linked chronic granulomatous disease (X-CGD) is an inherited disorder of the immune system caused by mutations in the *GP91PHOX* (*NOX2*) gene that regulates reactive oxygen species (ROS) production [72]. Wild-type *NOX2* was knocked into the *AAVS1* site of X-CGD patient-derived iPSCs, which can be derived to granulocytes exhibiting restored ROS production [72]. TALEN was also used to correct the mutation of beta-globin alleles in sickle cell disease (SCD) patient-derived iPSCs [73]. Together, these results suggest the effectiveness of TALEN-mediated gene editing in PSCs.

The specificities of TALENs were also investigated. In a parallel comparison of the ZFN and TALEN targeting *CCR6*, the TALEN exhibits lower off-target activity at the *CCR2* site [74]. Further study revealed that the ZFN and TALEN have different mutation signatures, as the TALEN induces significantly fewer insertions [75]. Off-target effects of TALENs can be further avoided by carefully choosing target sequences [76]. In PSCs, two studies employed WGS to confirm the off-target effects of TALENs in single-cell derived colonies [77,78]. As aforementioned, off-target rates at the population level of TALENs still await systematic evaluation with the new techniques (see the Section 2.3.1).

### 2.3. CRISPR-Cas System

Clustered Regularly Interspaced Short Palindromic Repeats (CRISPR)-Cas are bacterial and archaeal adaptive immunity systems that integrate segments of foreign nucleic acids into CRISPR arrays in host genomes [79,80]. Transcripts of these inserted segments (spacers) are employed as guide RNAs (gRNAs) to recognize and interfere with cognate targets (protospacers) [81]. The most important components of CRISPR-Cas systems are CRISPR RNAs (crRNAs) and Cas effectors [46]. With the assistance of crRNAs that recognize protospacer sequences, Cas effectors exhibit nuclease activities toward target DNAs or RNAs [46]. In the CRISPR-Cas9 system, which has been employed extensively in gene editing, another small RNA, the trans-activating crRNA (tracrRNA), is required [58,81,82]. The crRNA and tracrRNA form a double-stranded RNA which recruits the Cas9 protein to form a ribonucleoprotein (RNP) complex for target recognition and cleavage [81,83]. For the application, the crRNA and tracrRNA are further combined to the single-guide RNA (sgRNA) for ease of use [81]. One essential determinant for target recognition is the adjacent protospacer motif (PAM), the short DNA sequence next to protospacers [84]. For example, the PAM sequence for *Streptococcus pyogenes* Cas9 (*Sp*Cas9) is NGG [81]. The DNA cleavage induced by Cas9, like ZFNs or TALENs, triggers NHEJ or HDR repair pathways depending on the absence or presence of DNA templates, respectively, and results in indels or HR products. Thus, different from ZFNs or TALENs which recognize DNA targets by the protein–DNA interaction, the CRISPR-Cas systems utilize the nucleic acid base pairing as the mechanism to recognize their targets (DNA or RNA), greatly simplifying their design and application.

There are two distinct nuclease domains, HNH and RuvC, in Cas9, which cleave the target and non-target stand, respectively [81,83]. Inactivation of either HNH or RuvC domain creates the Cas9 nickase (nCas9), which can cleave only one DNA strand. If both HNH and RuvC domains are inactivated, the enzymatically dead Cas9, dCas9, could serve as a scaffold for recruiting effectors to the desired site without making DNA breaks. Depending on the factors fused with, dCas9 can be used for activating (CRISPRa) or suppressing (CRISPRi) gene expression, epigenetic modification (e.g., DNA methylation or histone modifications), or molecular imaging [85,86,87]. Although epigenetic regulations play key roles in PSC functions, applications excellently reviewed elsewhere are omitted in the present review due to the space limitation and the absence of permanent alterations of DNA sequences induced by these variants.

Except for Cas9, other Cas proteins, such as Cas3, Cas10, Cas12, Cas13, and Cas14, are also employed for other purposes based on their properties and substrates. For example, Cas3 and Cas10 cleave ssDNAs [88,89], Cas12 (including *As*Cas12a and *Lb*Cas12b from *Acidaminococcus* and *Lachnospiraceae* bacterium, respectively) cleaves both dsDNAs and ssDNAs [90], and Cas13 (including Cas13a and Cas13b) cleaves ssRNAs [91]. The diversity of the CRISPR-Cas system greatly expands the application repertoire, which is reviewed elsewhere [92]. Here, we focus on the utilization and off-target effects of the CRISPR-Cas9 system in human PSCs.

The CRISPR-Cas9 system has been extensively applied in human PSCs, although its off-target and side effects on genome integrity have not been extensively investigated yet. In addition to knocking out and knocking in specific genes, CRISPR-Cas9 has been employed for high-throughput screening using sgRNA libraries, which is less feasible for ZFNs or TALENs [93,94,95] (Figure 1). To build PSC-derived disease models, CRISPR-Cas9 usually performs genome engineering at the pluripotent stage, followed by differentiating PSCs into the desired cells/organoids. For example, CRISPR-Cas9 was applied to introduce *RBM20* mutations in human iPSCs, which were differentiated into cardiomyocytes to establish an in vitro model of dilated cardiomyopathy (DCM) [96]. CRISPR-Cas9 can also introduce inter-chromosome translocations to model blood cancers [97] (Figure 1). In addition to modeling disease, CRISPR-Cas9 was used to reverse disease mutations as exemplified by the correction of a diabetes-causing pathogenic mutant of *Wolfram syndrome 1* (*WFS1*) gene in iPSCs derived from a Wolfram syndrome (WS) patient. After transplantation, the genetically-corrected WS iPSC-derived β cells can reverse severe diabetes in mice [98].

Apart from 2D differentiation, human PSCs are also employed to build disease models or perform lineage tracing in organoids (Figure 1). Multiple organoids, including the brain, liver, retina, lung, blood vessels, heart, and kidney, have been generated from human PSCs [99,100,101,102,103,104]. For example, CRISPR-Cas9-mediated knockout of *PKD1* or *PKD2* in human ESC-derived kidney organoids can model polycystic kidney disease [105]. CRISPR-Cas9-mediated introduction of oncogenic mutations in human ESCs, which are then differentiated to cerebral organoids, can serve as a model to recapitulate brain tumorigenesis [106]. The introduction of the E50K mutation in optineurin (*OPTN*) by CRISPR-Cas9 has also been used to model glaucoma in ESC-derived retinal organoids [107]. To track hair cell induction during human inner ear organogenesis, CRISPR-Cas9 was used to construct *ATOH1* reporters in human ESCs, which are differentiated into inner cell organoids [108]. Recently, CRISPR-Cas systems have also served as DNA recorders and writers to trace sequential events during differentiation (see Conclusion and Future Prospects). Together, these results indicate the versatility of the CRISPR-Cas system in PSC applications (Figure 1).

CRISPR-Cas9-mediated gene editing has also been proposed for clinical cell therapies using human PSCs. For instance, cytokine-inducible SH2-containing protein (CISH) is a key negative regulator of interleukin-15 (IL-15) signaling in natural killer (NK) cells. Knockout of *CISH* in human iPSC-derived NK (iNK) cells by CRISPR-Cas9 improves their expansion capability, cytotoxic activity, and in vivo persistence to inhibit tumor progression in the leukemia xenograft model [109]. Knocking out the ectoenzyme *CD38* by CRISPR-Cas9 also improves in vivo persistence and antitumor activity of iPSC-derived NK cells in the absence of exogenous cytokine and elicits superior antitumor activity [110]. Thus, human PSCs could be a potentially unlimited, stable source of engineered “off-the-shelf” immune cells for cancer therapy (Figure 1).

#### 2.3.1. Evaluating Off-Target Effects of CRISPR-Cas9

As mentioned above, advanced methods have been developed to examine the off-target effects of programmable nucleases, especially CRISPR-Cas9. For example, Digenome-seq (digested genome sequencing), GUIDE-seq (genome-wide, unbiased identification of DSBs enabled by sequencing), HTGTS (the high-throughput genomic translocation sequencing), and BLESS (labeling, enrichments on streptavidin, and next-generation sequencing) can all identify DSBs generated by Cas9 in vitro or in vivo (reviewed in [111]). A parallel comparison between the ZFN, TALEN, and Cas9 targeting the same site demonstrated that Cas9 Is more efficient and specific than the other two, although whether it is universal for other target sites remains to be determined [112]. Despite its superior specificity, genome-wide analyses have shown that Cas9, like other programmable nucleases, can recognize and cleave DNA at off-target sites with sequences resembling on-target sites [113,114,115]. Employing a pair of nCas9 to create two single-strand breaks instead of one DSB can reduce the off-target rates, although it also compromises the editing efficiency [116]. Compared with other programmable endonucleases, the specificity of CRISPR-Cas9 can be and has been further improved by engineering Cas9 proteins. Mutations of the residues in Cas9 which are involved in Cas9-DNA interaction can reduce the binding of Cas9 at off-target sites, while the binding and editing ability at on-target sites are largely retained [117,118,119]. Modifications on the sgRNA, such as adjusting the length of the spacer region or adding secondary structures onto the 5′ ends of a sgRNA, also reduce off-target effects [113,120,121].

## 3. Base Editors

Since DSB-mediated editing raises concerns for undesired mutations in PSCs, base editors, which make base substitutions without introducing DSBs, are reasonably favored for gene editing in PSCs. Theoretically, six base editors will be needed for “any base to any base” substitutions (please refer to Chen et al. [125] for the illustration). Yet, pathogenic point mutations in humans are not evenly distributed (also Chen et al. [125] for the statistics), making it possible to cover most human diseases with fewer editors. The first two base editors reported, the cytidine base editor (CBE) and the adenine base editor (ABE), were realized by David Liu’s lab in 2016 and 2017, respectively [112,122]. CBE converts cytidines to thymines on one strand and thus can be used to create both C>T (C•T→T•A) and G>A (G•C→A•T) substitutions. Similarly, ABE substitutes adenines with guanines and thus can make both A>G (A•T→G•A) and T>C (T•A→C•G) substitutions. Those four types of editing cover ~60% of edits needed for correcting pathogenic mutations [125]. In 2020, two labs reported the glycosylase base editor that can make C>G and G>C conversions, which constitute ~10% of pathological mutations [123,124]. Recently, new editors were reported to be able to convert A to C or T [125,126], covering another ~25% of pathological mutations. In addition to these single-base editors, dual-base editors, which fuse two types of single-base editors, were also created to introduce multiple substitutions [127,128,129,130]. Together, these base editors constitute a tool collection for introducing or correcting point mutations in somatic or stem cells. In this section, we briefly review these single-base editors and discuss their strengths and remaining issues in PSC-based applications.

### 3.1. Cytidine Base Editor (CBE)

**Figure 2:**
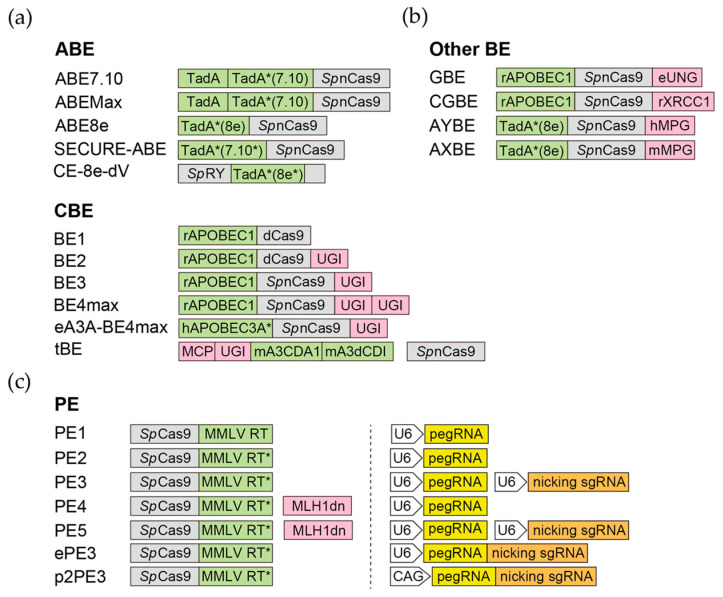
Base editors and prime editors. Representative versions of base editors and prime editors. The Cas9 derivatives are highlighted in grey, the key enzymatic components of BEs (**a**,**b**) and PEs (**c**) are highlighted in green, and the associated components are highlighted in pink. For PEs, the archi-tectures of promoters, pegRNAs (yellow), and nicking sgRNAs (orange) are also shown. The CAG promoter of p2PE3 can be replaced by other Pol II promoters.

CBEs take advantage of the cytidine deaminase activity, which converts cytidines to uracils (U), equivalent to T in base pairing, for base substitution. The rat APOBEC1 (apolipoprotein B mRNA editing enzyme, catalytic polypeptide-like 1, or rAPOBEC1) and the sea lamprey AID (activation-induced cytidine deaminase) were first employed in the BE series [131] and Target-AID [132], respectively. Cytidine deaminases from different species, including the CDA, AID, and APOBEC3 family, are also employed to build CBEs with different editing efficiencies and sequence preferences [133]. BE3 and BE4 are the third and fourth generation of BE, containing nCas9, rAPOBEC1, as well as one (BE3) or two (BE4) copies of uracil DNA glycosylase inhibitor (UGI) from the *Bacillus subtilis* bacteriophage [131] (Figure 2a). UGI can suppress the repair of U by inhibiting the uracil DNA glycosylase (UNG), which excises uracil bases to form abasic sites for base excision repair (BER). To date, more than thirty CBE variants have been made by changing the editing window, expanding the PAM compatibility, or increasing/decreasing their on-target/off-target activities [134]. Notably, despite its successful application in multiple cell lines, BE3 exhibits lower editing efficiency in human PSCs [135] and may need further optimization for better efficiency [136] (see the Section 3.5.2).

### 3.2. Adenine Base Editor (ABE)

In 2017, David Liu’s lab reported the first ABE, which realized the A>G and T>C substitutions [122]. Theoretically, deamination of adenosines results in inosines (I), which could complement with cytidines and be eventually converted to guanines by the mismatch repair. However, there are no known DNA adenine deaminases. To solve this issue, Gaudelli et al. subjected the *E. coli* tRNA adenine deaminase TadA to extensive evolution to alter its activity towards DNA [122]. ABE7.10, which contains a wild-type TadA and an evolved TadA* (contains 14 amino acid substitutions in the catalytic domain), was finally retrieved with the highest efficiency of converting A•T to G•C [122]. This breakthrough opens the gate for the subsequent derivation of ABE variants with improved nuclear localization/expression (ABEmax) [137] or smaller size and faster editing kinetics (ABE8e) [138]. Recently, several groups, including us, modified those ABEs to further reduce their off-targeting activities [139,140,141] or expand the PAM compatibility [142,143] (Figure 2a). Notably, compared with CBE, ABE exhibits high product purity and low rates of indels in mammalian cells, including human PSCs, possibly due to their lack of efficient glycosylase to initiate BER [122].

### 3.3. Glycosylase Base Editor (GBE) and C-to-G Base Editor (CGBE)

In 2020, three research groups reported base editors that can make C>G and G>C base transversions (purine > pyrimidine or pyrimidine > purine) in mammalian cells [123,124,135] (Figure 2b). All three base editors employ nCas9 and rAPOBEC1 (the same one CBE uses), while two of them (Kurt et al. [GBE] and Zhao et al. [CGBE]) replace the UGI with UNG and Chen et al. use XRCC1 protein instead of UGI. The UNG excises the U base created by the deaminase and generates the apurinic/apyrimidinic (AP) site that, in combination with the nick made by nCas9, initiates the DNA repair process (translesion synthesis) which favors insertion of G at the AP site. The C-to-G BE designed by Chen et al. uses XRCC1 to recruit BER proteins to repair the AP site created by endogenous UNG [135]. GBE and CGBE are further modified or optimized to relieve the PAM restriction, as well as to be more predictable and purer [144,145]. Recently, the Li lab reported a TadA-derived C-to-G base editor, Td-CGBE, which is based on an N46L variant of TadA-8e [146].

### 3.4. Adenine Transversion Editors (AYBE and AXBE)

As mentioned above, the A>C substitution is required to reverse ~25% pathological point mutations. Recently, two breakthrough studies reported base editors that can transverse A to C or T in mammalian cells [125,126] (Figure 2b). Both groups employ a strategy similar to GBE/CGBE, but aim at creating the AP site on A instead of C (in the GBE/CGBE case), which is expected to be mutagenized by the DNA repair pathway. To achieve this, Tong et al. constructed the adenine transverse base editor (AYBE, Y = C or T) by fusing the ABE8e with an engineered human hypoxanthine glycosylase enzyme, N-methylpurine DNA glycosylase (MPG, also called AAG). MPG excises the hypoxanthine group from the inosine produced by ABE, resulting in an AP site. The AP site will then be processed by the translesion synthesis pathway and replaced by C or T as the most common outcomes [126]. Chen et al. employed a similar strategy but used mouse AAG (MPG) instead of the human one, creating the AXBE. Importantly, by mutagenesis and Cas embedding strategies, Chen et al. further created the ACBE-Q editor, which exhibited high A>C activity and reduced A>G bystander substitutions [125]. Although the purity and efficiency of adenine transversion editors remain to be improved, these two editors have substantially expanded the potential of base editing.

### 3.5. Pros and Cons of Using Base Editors in PSCs

Although base editors cannot introduce indels or HR, they still possess wide applications in correcting point mutations, creating premature stop codons (knockout), and alternating splicing events. In contrast to DSB-dependent genome editing, base editors are considerably favored since most of them use nCas9, which only generates nicks. This advantage is particularly important for PSCs, in which the activation of p53-dependent DNA damage responses leads to detrimental consequences [33]. However, the deaminases may lead to undesired off-target edits on RNA or DNA in PSCs. Since off-target mutations on RNA could cause undesired phenotypes, and mutations on DNA will be transferred to all differentiated progenies, the off-target issue is one of the major concerns for applying base editors in PSCs. Here, we summarize the current knowledge on the off-target effects of base editors and the efforts researchers have made to resolve this issue.

#### 3.5.1. Dealing with Off-Target Effects of CBE and ABE

The rAPOBEC1 used by the BE series has off-target activities towards both DNA and RNA. Expressing BE3 in mouse embryos resulted in substantial off-target DNA single-nucleotide variants (SNVs) with more than 20-fold higher frequencies compared to CRISPR-Cas9 or ABE [147]. Most of the appeared mutations are independent of sgRNAs, suggesting that these off-target edits are attributed to the random, Cas9-independent binding of rAPOBEC1 to DNA [147]. A cohort study of BE3 transgenic mice also revealed five times more SNVs in the muscle tissue compared with the GFP control [148]. In contrast, ABE transgenic mice show barely detectable off-target DNA SNVs. The higher genome-wide off-target mutation rate of CBE over ABE is also observed in plants [149]. Although the off-target activity of the cytidine deaminase employed by the Target-AID system, pmCDA1, has not been evaluated by WGS, the R-loop assay on specific sites also suggests a high off-target activity on DNA [150]. Together, these results draw the attention to off-target DNA mutations generated by cytidine deaminases.

The off-target DNA activities of CBE and ABE in PSCs were also evaluated. In a well-controlled experiment, BE4 was tested for its off-target activities using syngeneic human iPSC clones with doxycycline-inducible CBE [151]. The WGS revealed that BE4 induces 10 times more SNVs compared with the control in human iPSCs. We also examined the off-target effects of overexpressed enhanced ABE (CE-8e-dV) in human ESC clones with a similar approach and did not observe mutations beyond the background level [143]. These results confirm that early version CBEs exhibit higher off-target rates on DNA compared with ABE in PSCs. Notably, we only uncovered a few (<50) differentially expressed genes upon CE-8e-dV overexpression, and no activation of the p53 signaling pathway was observed, further indicating the safety of ABE in PSCs [143].

The off-target activity on RNA is an even more important issue for rAPOBEC1-based CBE. Grünewald et al. performed RNA-seq and WGS on BE3-overexpressed human cells. On the transcriptome-wide scale, they uncovered tens of thousands of C>U edits with frequencies ranging from 0.07–100% in 38–58% of expressed genes, which resulted in missense, nonsense, splice site, 5′ UTR, and 3′ UTR mutations [152]. The parallel WGS confirmed that most of those RNA mutations are not originated from DNA [152]. In contrast to the nature of rAPOBEC1 that targets both DNA and RNA, DNA is likely the only natural substrate for AID [153]. For ABE, despite its minimal off-target DNA editing activity, researchers also found ABE generates lower but evident A>I editing in cellular RNAs, possibly due to the deaminase activity of TadA/TadA* towards RNA [140]. Our study in human ESCs also revealed RNA off-target activity of ABE8e [143]. Together, these results suggest diverse influences on the transcriptome by CBE and ABE in PSCs.

Many efforts have been made to resolve the off-target issue of CBE and ABE. For CBE, mutations were introduced to cytidine deaminases to segregate its activity towards DNA and RNA. For example, the Joung group engineered two SECURE-bEs by introducing R33A or R33A/K34A to rAPOBEC1, which greatly reduces the C>U editing on RNA while maintaining the on-target efficiency on DNA [152]. They also replaced rAPOBEC1 with an engineered human APOBEC3A (hA3A) domain in the BE3 system, which can perform base editing in the CpG context with low off-target rates [154]. The Liu and Yang groups found that W90Y/R126E mutations in rAPOBEC1 (YE1) greatly reduce the off-target activity of BE3 on DNA and RNA [155,156,157]. Wang et al. constructed a transformer BE (tBE) system by fusing a cleavable deoxycytidine deaminase inhibitor (dCDI) domain to cytidine deaminases, resulting in efficient editing with only a background level of off-target mutations in the whole transcriptome and the genome [158] (Figure 2a). Recently, three groups reported TadCBE, CBE-T, and Td-CBE, all of which use engineered/evolved TadA (the ABE component) to perform cytidine deamination with the advantages of high on-target activities, smaller sizes, and substantially lower DNA and RNA off-target activities [146,159,160].

Similar to CBEs, ABEs were also engineered for better on- and off-target performance on both DNA and RNA. Liu and other groups employed different strategies to further reduce off-target mutations induced by adenosine deaminases, including the point mutation V106W [140,161], deletion of the key residue R153 [162], and embedding the editing enzymes into the middle of nCas9 to hamper their access to off-targets [139]. We combined those three strategies to make a new ABE, CE-8e-dV, and tested its performance in human ESCs [143]. By WGS and RNA-seq analyses, we confirmed that CE-8e-dV exhibits background-level DNA off-target effects and only ~1/3 off-target RNA edits compared with ABE8e [139]. Chen et al. reported that introducing the L145T mutation to ABE8e (ABE9) could further decrease its editing window and bystander editing [163]. Finally, mutagenesis-based engineering also enables researchers to create ABE9e (R111T/N127K/Q154R), which has a significantly lower bystander mutation rate in human ESCs [164].

#### 3.5.2. On- and Off-Targets of Other Base Editors in PSCs

GBE, CGBE, AYBE, and AXBE are newly developed base editors. The efficiency of CGBE has been examined in human H9 ESCs: both CGBE and BE3 exhibit low editing efficiency in H9 cells, which might be due to specific methylation profiles in stem cells that inhibit editing [135]. Although AXBE has been employed in mice and human cells [125], its activity in human PSCs remains to be evaluated since linking hAAG to ABEmax or ABE8e (same as the AYBE design) does not result in A>Y transversion in PSCs [165]. In the aggregate, the on-target efficiencies of C>G and A>Y editors in human PSCs still await examination by transcriptome- and genome-wide analysis. Finally, overexpression of some components of editors, such as XRCC1 or MPG, could also have unexpected influences on PSCs and needs to be addressed.

## 4. Prime Editor (PE)

In 2019, a versatile gene-editing tool, prime editor (PE), was developed by the Liu lab [166,167]. The two essential components of the PE system are the editor, a nCas9 fused with reverse transcriptase (RT), and a single engineered prime editing guide RNA (pegRNA) which consists of the sgRNA and the intended sequence to edit. After being guided to the target site by the sgRNA component of the pegRNA, the nCas9 generates a nick in the single-stranded R-loop of the target site. The pegRNA then hybridizes with the nicked target DNA strand and serves as the template for RT to polymerize the desired sequence onto the nicked target DNA. After resolving the flap of the edited DNA by DNA repair machinery, the desired sequence will be incorporated into the genome. Decided by the templates, PE can make all types of base substitutions or insertions/deletions of small DNA fragments in mammalian cells [166,167].

PEs have evolved through multiple versions (Figure 2c). The original version of prime editor (PE1) uses the wild-type RT from Moloney murine leukemia virus (MMLV) [166]. Although PE1 can make all gene edits in human cells, the gene editing efficiency is low, typically <5% [166]. In PE2, five mutations are introduced to MMLV-RT to enhance its thermostability, processivity, and binding affinity to the template. The gene editing efficiency of PE2 is increased 1.6- to 5.1-fold compared to PE1 in human cells [166]. On the basis of PE2, PE3 includes an additional sgRNA to direct the nCas9 component of the prime editor to also nick the non-edited strand, promoting the replacement of the non-edited strand with the sequence complementary to the edited DNA [166]. The gene editing efficiency of PE3 is further increased 1.5- to 4.2-fold compared to PE2 in HEK293T cells [166]. PE4 and PE5 prime editing systems are developed by the transient expression of an engineered mismatch repair (MMR)-inhibiting protein, MLH1dn, with PE2 and PE3, respectively. The rationale behind this design is that the MMR was found to strongly antagonize prime editing and promote the generation of undesired indel byproducts [168]. Compared with PE2 and PE3 systems, PE4 and PE5 prime editing systems enhance the editing efficiency by an average of 7.7- and 2.0-fold, respectively [168]. PEmax, which contains R221K/N394K mutations in Cas9, two NLS (nuclear localization signal) tags, and a codon-optimized MMLV-RT, exhibits further elevated editing efficacy [168]. Finally, replacing the nCas9 with the DSB-making Cas9 also significantly enhances the editing efficiency [169,170,171,172]. Notably, since DSBs are made by late versions of PE (since PE3), their side effects need to be determined, particularly in PSCs.

The PE system was also modified or optimized by researchers from the perspectives of editors or pegRNAs. The modification on editors is mostly by fusing with other proteins to enhance the editor’s performance. In the hyPE2 design, the Rad51 DNA-binding domain is inserted between nCas9 and RT to facilitate reverse transcription [173]. Fusion of the chromatin-modulating peptide to PE3 (CMP-PE3) or a DNA repair-related peptide to PE2 (IN-PE2) can significantly increase the editing efficiency in mammalian cells [174,175]. On the other hand, the pegRNA design is optimized by various rationales. The pegRNA contains a primer binding site (PBS) sequence to trigger the reverse transcription, whose length greatly affects the editing efficiency [176]. Several algorithms or approaches were developed to optimize the PBS length or the pegRNA sequence [177,178,179,180,181,182,183]. Modifying the pegRNA by stabilizing its secondary structure or preventing its circularization also enhances the editing efficiency [176,183,184,185,186].

The efficiencies of PEs vary widely, depending on the genomic context, the pegRNA design, and the cell type. In our work, PE editing efficiencies on the same sites are consistently lower in PSCs compared with immortalized cells, such as HEK293T [187]. The causes of such differences remain unclear. One possibility of the low efficiency could be simply the level of PE/pegRNA expressed in cells (see Conclusion and Future Prospects). The high MMR repair capacity of PSCs could also result in this low editing efficiency, as overexpressing MLH1dn (PE4/5) enhanced the editing efficiency of PE in PSCs [187]. Interestingly, inhibition of p53 by SV40 large T antigen (SV40LT) further increases the editing efficiency, suggesting p53 plays a role in modulating PE-mediated edits [187]. Despite the involvement of p53, our and other researchers’ results suggest that PE-editing, in the presence or absence of editing boosters (MLH1dn or SV40LT), does not lead to off-target mutations beyond the background level [187,188].

Although the efficiency remains to be improved, prime editing has been successfully applied for gene editing in human PSCs to induce nucleotide substitutions or small insertions/deletions. Habib et al. used PE to correct a liver disease-related mutation of *SERPINA1* in patient-derived human iPSCs. PE was also used to precisely delete the intronic splicing silencer-N1 (ISS-N1) within survival motor neuron 2 (*SMN2*) to rescue full-length SMN expression in human iPSCs derived from spinal muscular atrophy (SMA) patients [189]. Finally, Li. et al. reported that delivering PE and pegRNA in the mRNA form greatly enhances the editing efficiency on multiple sites, which greatly facilitates applying PEs in human PSCs [190]. However, whether this approach can generally increase the efficiencies of PEs on different target sites remains to be investigated. Finally, compared with the base editors (CBEs or ABEs), PEs exhibit lower efficiency but fewer bystander edits. Importantly, the WGS confirmed that PE does not lead to off-target mutations in the genome in PSCs [188]. Together, these results suggest that PEs are promising editing tools to for PSCs, although their caveats remain to be solved.

## 5. New Gene Editing Tools

Although DSB-independent editing tools currently used are favored in PSCs, one major restriction of those tools is the inability or low efficiency to insert large DNA fragments. PEs can insert short DNA fragments (<20 nt), yet their efficiencies drop dramatically with the increased length of insertion [187]. This property does not meet the need of many clinical applications (e.g., CAR-iNK), which call for much larger insertions. Currently, several new gene editing tools have been developed for knocking in larger DNA fragments (see Section 5.1, Section 5.2 and Section 5.3) or to enrich cell populations containing these insertions (see Section 5.4). Of note, some of these tools are still under development and need improvements for applying in mammalian cells or PSCs.

### 5.1. CRISPR-Associated Transposon (CAST)

As the name suggests, the CRISPR-associated transposon (CAST) is the transposon containing a specific subtype of CRISPR-Cas systems [191]. Compared with RNA-guided endonucleases that function in the defense against MGE (mobile genetic elements), this specific CRISPR-Cas subtype is employed for RNA-guided transposition [192]. The mechanisms of two types of CASTs, CAST I-F and CAST V-K, were elucidated in prokaryotes [193]. As the recognition–integration process is independent of HDR, transposon-based CRISPR systems hold great expectation for inserting large DNA fragments into specific sites in eukaryotic cells. Recently, a system based on CAST, the HE-assisted large-sequence integrating CAST-complex (HELIX), has been able to insert DNA fragments into exogenous plasmids in human cells [194]. Furthermore, Lampe et al. reported that with the help of bacterial ClpX, Type I-F CAST could reach single-digit efficiencies in human endogenous genes [195]. Despite this success, CAST is still ineffective in editing human endogenous genes, possibly due to the unidentified regulatory factors, components, or features of eukaryotic chromatin. More understanding of structures and mechanisms of CASTs could facilitate their application in eukaryotic cells, even PSCs.

### 5.2. CRISPR-Associated Serine Recombinases (twinPE and PASTE)

Another strategy to integrate large DNA fragments is using recombinases, which integrate MGE into bacterial genomes on attachment sites, the specific sequences into which the payloads will be inserted. Recently, thousands of large serine recombinases (LSRs) and DNA attachment sites were predicted using computational approaches, and over 60 new LSRs were experimentally validated in human cells [196]. In combination with TwinPE, which exhibits superior ability to insert the landing pad sequence into the desired site, large DNA fragment insertion can be mediated by the site-specific serine recombinase/integrase, Bxb1 [197]. Another approach with a similar concept, PASTE (programmable addition via site-specific targeting elements), uses the PE-Bxb1 fusion protein and the pegRNA containing the attachment sequence (atgRNA for attachment site-containing guide RNA) [198]. Both twinPE and PASTE can insert large DNA fragments ranging from 5.6~36 kb into human immortalized cells or cancer cells, sufficient for most purposes. It will be valuable to test the efficiencies of those tools in PSCs.

### 5.3. Retron

Retrons are non-transposable retroelements firstly identified in prokaryotes [199]. A typical retron contains a reverse transcriptase (RT) and a template sequence, on which the RT acts to create the multi-copy single-stranded DNA (msDNA) [200,201]. The msDNAs are then joined to their template RNAs by a 2′–5′ phosphodiester bond, forming a special DNA–RNA hybrid structure [201]. Although the functions of retrons in their hosts remain poorly understood [202], the retron scaffold has been modified for the purpose of genome editing: a sgRNA can be added to the RNA component of the retron to guide it to the target site in the presence of Cas, while the desired donor sequence can be inserted into the retron scaffold and retro-transcribed into the msDNA [203]. As the consequence, those msDNAs containing desired sequences will be enriched in the proximity of the sgRNA-guided cleavage site and be used as the donor template for HR [203]. This system, named CRISPEY (Cas9 retron precise parallel editing via homology), has been employed in yeasts for massive parallel genome editing [203]. Recently, retrons have been successfully applied for gene editing in mammalian cells, although their efficiencies remain low [204,205]. Other concerns for the retron system are its DSB-dependent HDR mechanism and the limited fragment size (~700 bp) that can be inserted into the retron framework. Together, more mechanistic studies are required to apply retrons in mammalian cells.

### 5.4. SeLection by Essential-Gene Exon Knock-in (SLEEK)

Precise knock-in of genes at desired, endogenous sites is required for many clinical purposes. However, the desired knock-in mediated by CRISPR-Cas9-induced HDR is usually mixed or even overwhelmed by undesired indels generated by NHEJ. Recently, a simple but efficient approach to enrich cells with correct knock-in was developed. In SLEEK (selection by essential-gene exon knock-in), the donor DNA fragment is targeted to a site within an exon of an essential gene. The cargo template is designed in a way that the correct knock-in will retain the essential gene function, while all the cells containing undesired products get wiped out without the need for drug selection [206]. Importantly, this method has been applied in iPSCs to knock-in *CD16* and *mbIL-15*, which enhance the anti-tumor activity and persistence of iNK cells [206]. Although the WGS of those iPSC clones is still needed to evaluate the consequences of DSB-dependent HDR, this method provides a great advantage in saving the cost of generating clinical level PSCs.

## 6. Conclusions and Future Prospects

### 6.1. Improving Editing Efficiencies in Human PSCs

Considering the laborious process to isolate and characterize single-cell derived colonies, improving the gene editing efficiencies is crucial to apply gene editing tools in human PSCs. The Doudna and Church groups demonstrated that Cas9-mediated gene editing in PSCs is less efficient than in other somatic cells [82,207]. Previous studies also reported decreased editing efficiencies of CBE and PE in human PSCs [208]. However, it is over-simplistic to directly compare the editing efficiencies between somatic/immortal cells and PSCs. Human PSCs are notoriously hard to deliver exogenous genes with high copies. Thus, the low editing efficiencies of editors in PSCs could be simply due to their low expression levels compared to cell lines that can be easily transfected. In agreement with this notion, recent studies suggested that PE delivered in the mRNA form greatly enhances the editing efficiency compared to other forms, such as plasmids or RNP complexes [190]. Recent studies also showed that delivering Cas RNPs (Cas9 or Cas12) by cell-penetrating peptides greatly enhances editing efficiencies in human T cells or hematopoietic progenitor cells [209,210]. It is anticipated that the editing efficiencies of base editors or PEs in human PSCs can also be improved with these delivery techniques.

In addition to modifications on editors or gRNAs, small molecules were also found to be able to manipulate editing efficiencies. Small molecules that can enhance the HDR activity, such as L755507 and Brefeldin A, also increase CRISPR-mediated HDR efficiencies in mouse ESCs and human non-pluripotent cells [211]. Inhibitors targeting key components of NHEJ also increase the HDR rate in human non-pluripotent cells and mouse embryos [212,213,214]. In addition, inhibition of ATM or ATR could enhance both knockout and knock-in efficiencies of Cas12a (Cpf1) in human PSCs, although whether it is applicable for Cas9 remains to be determined [215]. Finally, histone deacetylase (HDAC) inhibitors can enhance Cas9-, CBE-, and ABE-mediated editing by increasing both the expression level of proteins and target accessibility in human non-pluripotent cells [215]. Investigating these boosters and their influences on off-target effects as well as genome integrity in the context of human PSCs will benefit future applications.

### 6.2. Conditional Gene Editing

The major concern of performing gene editing in PSCs is that off-target edits will be carried to their differentiated progenies. One potential solution of that is to construct inactive editing components in PSCs which will be activated upon differentiation to perform editing in somatic (stem) cells that have limited longevity. This design can be achieved by putting editors and/or gRNAs under the control of specific promoters. However, most RNA polymerase III promoters used to drive gRNA expression are constitutively active. We recently established a novel PE, p2PE3, using RNA polymerase II promoters to drive the expression of pegRNA and sgRNA [187] (Figure 2c). The p2PE3 displays 2.1-fold higher editing efficiency compared to PE3, and can be combined with SV40LT and/or MLH1dn to further increase its editing efficiency in human PSCs [187]. Using this system, the PE and pegRNA can be integrated as a cassette and put under the control of drug-inducible or lineage-specific promoters. This conditional editing strategy can also be employed by Cas9 or base editors to avoid undesired mutation in PSCs.

### 6.3. CRISPR-Cas as DNA Recorders of Cell Fates

PSCs are reliable in vitro models for differentiation, which involves progressive transitions of cellular states. General lineage tracing approaches only allow marking one or two states (e.g., the Cre or the Cre/Dre dual system). Recently, the CRISPR-Cas system has been employed to record signaling, cellular, or transcriptional events on DNA of eukaryotic cells (i.e., using DNA as the memory device) [216,217,218,219]. Among them, the “DNA typewriter” technique employs an elegant design, using sequential prime editing to capture different events in the happening order [219]. With this technique, different events, such as transcription activation or signal transduction, can be encoded by different pegRNAs driven by specific promoters (i.e., the p2PE3 system mentioned before) built in PSCs. Upon 2D or 3D differentiation, the order of happened events can be recorded on the “DNA tape” in each cell and decoded by single-cell sequencing. This system could be a unique tool to reveal complex event histories during cell fate specification.

### 6.4. New Systems to Be Explored

In nature, there are still broad varieties of RNA-guided nucleases, transposases, and recombinases that remain unexplored and can be potentially employed as gene editing tools. For example, the Doudna group identified the CIRSPR-CasΦ system from the Biggiephage [220], which is only half the size of Cas9 and has been employed for gene editing in plants [221]. A more thorough investigation identified ~6000 phage CRISPR-Cas systems covering all six known CRISPR-Cas types [222]. One of them, Casλ, was characterized and able to perform gene editing in HEK293T cells [222]. By tracing the ancestor of Cas proteins, the Zhang and Siksnys groups identified three IS200/IS605 transposon-encoded proteins, IscB, IsrB, and TnpB, which are also RNA-guided DNA nucleases [223,224]. Both IscB and TnpB exhibit gene editing activity and can be incorporated in base editors with high efficiencies in human cells [223,224,225,226]. Surprisingly, two very recent studies suggest that TnpB homologs are widespread in eukaryotes [227,228]. Saito et al. and Jiang et al. characterized the RNA-guided DNA nuclease activity of the eukaryotic transposon-encoded Fanzor proteins. Both studies demonstrated that Fanzor proteins from different species can be reprogrammed for human genome engineering in HEK293T cells [227,228]. Those eukaryotic RNA-guided endonucleases not only have hypercompact sizes but also exhibit low cleavage activity on collateral nucleic acids. Since they are originated from eukaryotic cells, Fanzor proteins are expected to have great application potential in the future.

In addition to novel nucleases, the Zhang group also elucidated the transposition mechanism of a non–long terminal repeat (non-LTR) retrotransposon, the R2 retrotransposon. Non-LTR transposons are inserted into genomes by a mechanism called target-primed reverse transcription (TPRT), during which the target DNA sequence is nicked, priming the reverse transcription of retrotransposon RNA. The Zhang group resolved the structure of the silk moth R2Bm (LINE type) TPRT complex and elucidated the mechanism of how R2Bm recognizes its native target to initiate TPRT [229]. Importantly, the Zhang group found that Cas9 can retarget R2 in vitro and initiate TPRT [229]. Although the integration events have not yet been observed in vitro or in vivo, this finding suggests its future use as a site-specific insertion tool.

### 6.5. Conclusions

With the optimization of current tools and the discovery of new tools, it is predictable that “safe” gene editing in PSCs will be easier to perform in the future. Notably, since the specificity of gene editing in PSCs has to meet the highest criteria, those gene editors validated in PSCs can also be potentially applied to other gene- or cell-therapies. Undeniably, current PSC-based therapies still face concerns, such as the removal of residual undifferentiated cells as well as the immunogenicity and under-performance of PSC-derived cells. Gene editing could also be employed to resolve those issues by engineering cells for lower immunogenicity (e.g., removal of HLA) or high efficacies/functions (e.g., expression of stimulatory or sustaining cytokines). Considering PSCs’ unique merits of stability and unlimited quantity, it is worthy to further develop and validate gene editing tools for PSCs to facilitate their applications in both basic and translational research.

## Figures and Tables

**Table 1:** Multifaceted comparison of gene editing tools.

	DSB-Dependent Editor			Base Editor				Prime Editor
	ZFN	TALEN	SpCas9	CBE	ABE	CGBE	AXBE/AYBE	
Type of DNA damage	DSB	DSB	DSB	SSB	SSB	SSB	SSB	SSB (PE2) or DSB (PE3)
Type of editing	Indel; Knock-in; Base mutation /correction	Indel; Knock-in; Base mutation /correction	Indel; Knock-in; Translocation; Base mutation /correction	Base substitution	Base substitution	Base substitution	Base substitution;	Base substitution; Indel; Recombination
p53 activation?	Yes	Yes	Yes	No	No	N/A	N/A	No
On-target specificity	+	+	++	++	+++	++	+ (C/T mix)	+++
Off-target effects on DNA	++	++	++	++	Very low	++ (based on CBE)	Very low (based on ABE)	Low
Off-target effects on RNA	-	-	-	++	+	++ (based on CBE)	+ (based on ABE)	-
Applied in human PSCs?	Yes	Yes	Yes	Yes	Yes	No	No	Yes
Clinical trial?	Yes	Yes	Yes	Yes	Yes	No	No	No
